# Possible Impact of Lymphatic Drainage on Brain Injury After Aneurysmal Subarachnoid Hemorrhage

**DOI:** 10.3390/ijms27031329

**Published:** 2026-01-29

**Authors:** Hidenori Suzuki, Koichi Hakozaki, Kazuaki Aoki, Fumihiro Kawakita, Yoshinari Nakatsuka, Yotaro Kitano, Hirofumi Nishikawa, Ryuta Yasuda

**Affiliations:** Department of Neurosurgery, Mie University Graduate School of Medicine, 2-174 Edobashi, Tsu 514-8507, Mie, Japan; hakozakik@med.mie-u.ac.jp (K.H.); 322d001@m.mie-u.ac.jp (K.A.); fumiwo0219@med.mie-u.ac.jp (F.K.); info@nobonoclinic.jp (Y.N.); ykitano@med.mie-u.ac.jp (Y.K.); hnishikawa0220@med.mie-u.ac.jp (H.N.); ryasuda@med.mie-u.ac.jp (R.Y.)

**Keywords:** delayed cerebral ischemia, early brain injury, lymphatic drainage, matricellular protein, subarachnoid hemorrhage

## Abstract

Subarachnoid hemorrhage (SAH) due to ruptured cerebral aneurysms is the most severe form of stroke, and treatment outcomes remain poor. Brain damage after SAH can be broadly divided into early brain injury (EBI) and delayed cerebral ischemia (DCI). Although the causes of these events are multifactorial, free hemoglobin generated after hemolysis in the subarachnoid space is believed to be one of the most important causative factors. Recently, cerebral lymphatic vessels, previously thought to be non-existent, have been identified, suggesting their involvement not only in maintaining homeostasis but also in brain injury. Furthermore, new findings have been reported regarding cerebrospinal fluid (CSF) circulation. Because intracranial CSF circulation and lymphatic drainage to the extracranial blood and lymphatic vessels affect free hemoglobin metabolism in the CSF, these factors are likely to affect EBI and DCI. In addition, matricellular protein tenascin-C, which we have reported to be involved in the pathogenesis of EBI and DCI, has been reported to inhibit lymphatic vessel proliferation in non-central nervous system pathologies. However, the relationship between post-SAH brain injury and intracranial lymphatics remains unknown. This review aimed to summarize recent findings regarding intracranial lymphatics and CSF circulation and to discuss how they may affect post-SAH pathology.

## 1. Introduction

Subarachnoid hemorrhage (SAH) due to the rupture of an intracranial cerebral aneurysm still has a poor outcome and is accompanied by severe brain damage [1]. The causes of brain damage can be broadly divided into transient global cerebral ischemia caused by increased intracranial pressure (ICP) at the time of aneurysm rupture and a series of pathological reactions induced by blood components released into the subarachnoid space, and the latter is currently the main treatment target [1]. Although the pathophysiological mechanism of brain damage after SAH is complex and multifactorial and has not been fully elucidated, free hemoglobin (Hb) is believed to be one of the most upstream and important inducers of underlying pathologies [1,2,3]. The pathologies include neuroinflammation, cerebral arteriolar or arterial spasm, blood−brain barrier (BBB) disruption, microthrombosis and neuroelectric disorders, leading to early brain injury (EBI) in the acute phase and delayed cerebral ischemia (DCI) in the delayed phase [1,2,3].

In SAH, glymphatic and meningeal lymphatic functions are impaired, preventing effective drainage of toxic substances such as free Hb from the cerebrospinal fluid (CSF) space into the extracranial blood and lymphatic vessels, which may worsen EBI and DCI [4,5]. Therefore, a rational strategy would be to allow free Hb to be removed from the intracranial space with little adverse effect before the cascade of reactions is initiated. In a clinical setting, early blood removal has been tried by means of direct surgery, irrigation and CSF drainage with or without intrathecal thrombolysis, but the efficacy has been limited [3]. Recently, new findings have been discovered regarding the glymphatic system and the extracranial CSF drainage mechanism, which are expected to help elucidate the pathology of various diseases and be applied to their treatment [4,5,6]. Although knowledge about the intracranial lymphatic pathway is still limited, this article summarizes research findings on intracranial lymphatic drainage over the past 10 years and discusses expectations for future developments, focusing particularly on their application to the treatment of SAH. Although the glymphatic system and meningeal lymphatic drainage have been extensively studied in animals, particularly rodents, investigations of the human system have primarily relied on magnetic resonance imaging (MRI) and autopsy findings. Therefore, findings on the human glymphatic system and meningeal lymphatic drainage obtained from MRI and autopsy are summarized in Section 7 and Section 8.

## 2. Intracranial Lymphatic Vessels

Lymphatic vessels are not only involved in maintaining homeostasis through the absorption of interstitial fluid (ISF) and the collection of immune cells, but also function as endocrine organs with secreted proteins involved in tissue damage [7]. Recently, brain lymphatic vessels, which were previously thought to be non-existent, have been identified, suggesting their involvement in physiological functions and diseases, as well as immune tolerance in brain [8]. Conventionally, treatment of EBI and DCI after SAH has focused on controlling ICP and suppressing a series of pathological reactions [9]. However, controlling lymphatic drainage to the extracranial blood or lymphatic vessels may lead to the development of new treatments. Our recent study demonstrated that post-SAH intrathecal administration of a clinically available Hb scavenger protein haptoglobin (Hp), which irreversibly binds to free Hb and forms Hp–Hb complexes to prevent Hb’s autooxidation and toxicity, inhibited not only cerebral artery spasm in the subarachnoid space, but also EBI in terms of the activation of microglia, formation of microthrombus, development of neuronal apoptosis and brain edema in mouse SAH models [10]. The formation of Hp–Hb complexes resulted in large molecules that were unable to enter the paravascular spaces in the brain parenchyma, suppressing the intraparenchymal Hb infiltration and promoting Hb clearance to the deep cervical lymph nodes via lymphatic drainage [10]. These findings support that maintaining or improving lymphatic drainage, which is impaired after SAH [4], is important for reducing post-SAH Hb toxicity.

## 3. The Fate of Free Hb After SAH

**Figure 1:**
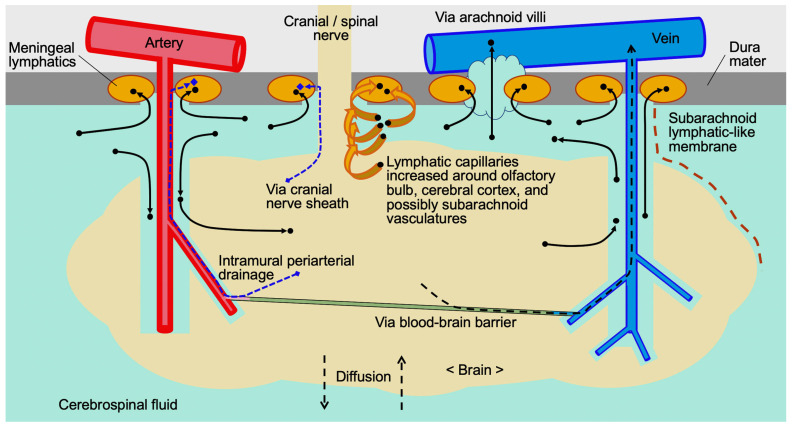
Assumed circulation of cerebrospinal and intraparenchymal interstitial fluids and lymphatic drainage. After subarachnoid hemorrhage (SAH), lymphatic drainage and the glymphatic system are impaired and, as a compensatory mechanism, lymphatic capillaries may increase around the olfactory bulb, cerebral cortex and subarachnoid vessels. ●, free hemoglobin after SAH; ◆, lower molecular weight macromolecule; dotted arrow, fluid and solute.

Although the exact mechanism by which red blood cells (RBCs) and free Hb extravasated into the subarachnoid spaces are removed remains unclear, their clearance mechanisms have been thought to involve phagocytosis and excretion together with the CSF [11]. After SAH, a small fraction of extravasated RBCs in the subarachnoid space may leave the intracranial space prior to RBC lysis [12]. However, most of the extravasated RBCs are either lysed osmotically by activated complement and the other mechanisms to release free Hb or undergo phagocytosis, which is carried out by CSF- or blood-derived meningeal and perivascular macrophages and neutrophils via the scavenger receptor CD36 over the course of days [11,13,14]. Erythrophagocytic cells detoxify Hb by heme oxygenases with an iron chelator ferritin and exit the intracranial space either by migrating along cranial and spinal nerves and through meningeal lymphatic drainage, or by being directly transported to the vasculature through arachnoid villi, disrupted BBB or the choroid plexus blood−CSF barrier (Figure 1) [12,15]. On the other hand, hyperphagic macrophages that phagocytose two or more RBCs result in cell death, releasing toxic heme and iron into the extracellular space [10,15]. The released heme is sequestered by hemopexin, and the hemopexin−heme complexes are endocytosed by macrophages, pericytes, smooth muscle cells, endothelial cells, astrocytes and neurons via the low-density lipoprotein receptor-related protein-1/CD91 receptor: the endocytosed heme is also degraded by heme oxygenases [15,16]. However, hemopexins in the CSF are scarce, although most (about 90%) of them are synthesized in glial cells and neurons under healthy conditions [15,16].

Hb exists in RBCs as a tetramer, but after hemolysis released free Hb changes from a tetramer to a dimer (32 kDa) in the CSF [10]. Free Hb dimers distribute across tissue barriers such as the pia mater, ependymal cell layer and glia limitans, penetrating deep into the brain tissue via the paravascular spaces as well as into the arterial wall to induce a series of pathological reactions [10]. Free Hb dimers are scavenged by Hp that irreversibly binds free Hb to prevent its autooxidation, but the amount of Hp in the CSF is extremely small [13,15]. Hp is normally produced in the liver and reticuloendothelial system and barely diffuses into the CSF: under pathological conditions such as SAH, Hp may increase in oligodendrocytes, astrocytes and neurons but remain deficient [13,15,16]. In SAH, macrophages, microglia and neurons endocytose the Hp–Hb complex via the scavenger receptor CD163, but neurons that take up the complex undergo cell death [13,15,16]. Free Hb dimers also bind to soluble CD163 and immunoglobulin G (IgG) to form soluble CD163−Hb−IgG complexes, which are endocytosed by microglia and microphages via the crystallizable fragment-γ receptor [13,16]. Free Hb dimers that are not scavenged also release heme, only a small portion of which is sequestered by hemopexins [16].

## 4. The Role of the Choroid Plexus

The choroid plexus is a highly vascularized structure that acts as a gateway for CSF production and circulation and immune surveillance of the central nervous system (CNS), thereby participating in the glymphatic system for waste removal [17,18]. More than 50% of CSF is produced in the choroid plexus, and the remainder comes from ISF produced by the BBB and the ependymal cells lining the ventricles [17]. Because CSF production in the choroid plexus is the driving force behind glymphatic flux, it has been thought that the CSF production rate and glymphatic clearance rates are consistent [17]. It is known that the function of the choroid plexus is impaired by aging and disease [17]. After experimental SAH, it was reported that blood degradation products and increased ICP may induce an inflammatory response in the choroid plexus, which not only appears to promote Hb metabolism but may also lead to neuroinflammation, DCI and hydrocephalus [19,20].

## 5. Possible Intracranial CSF and Hb Circulation

Meningeal lymphatics have no direct contact with brain parenchyma under physiological conditions [21]. In the brain, the circulation, exchange and recycling of fluids are mediated by the glymphatic system [21]. In the glymphatic system, CSF enters the brain parenchyma along arteries and arterioles from the subarachnoid space by arterial and arteriolar pulsation, is filtered through the brain interstitial spaces and is drained along venules and veins [22,23,24]. RBCs (diameter, 7–8 μm), free Hb tetramers (about 64.5 kDa), fibrinogens (about 340 kDa) and antibodies (IgG monomer, about 150 kDa) can penetrate from the subarachnoid space into the intraparenchymal paravascular space of arterioles and capillaries and can also flow back into the intraparenchymal paravascular space of venules when ICP is high [1,13,15,25]. Substances ≤ 100 kDa can pass through the 50 nm clefts between astrocytic endfeet into the brain interstitial space, while the passage of substances > 200 kDa is restricted [13,15]. The molecular mass of Hp–Hb complexes varies from the simplest dimeric form (162 kDa) below the threshold to the highest-order polymer (1760 kDa) [13,15]. Convection between CSF and ISF can occur along perivascular and paravascular pathways, along white matter tracts surrounding the ventricles and by simple diffusion through the interstitial spaces of the gray matter (Figure 1) [23,26].

CSF drains into meningeal lymphatics in response to ICP and then travels via two routes to deep cervical lymph nodes: one adjacent to the jugular vein and the other through the nasopharyngeal lymphatic plexus, which join just before the lymph nodes [24]. Drainage to deep cervical lymph nodes occurred spontaneously in the absence of lymphatic pumping and was linked to ICP, whereas drainage to superficial cervical lymph nodes was driven by pumping [24]. Fluid outflow also occurs via the cribriform plate, although this particular pathway may be less active in humans than in rodents and occurs along other nerve and vascular exit points as well, including the spinal column, ultimately leading to drainage via other lymphatic networks [24]. Lymph flows in meningeal lymphatics are increased to drain extravasated RBCs, Hb, other macromolecules and immune cells from CSF into cervical lymph nodes after SAH [10,11,27]. Although fluid and solutes may diffuse into lymphatic vessels due to the pressure difference between the ISF or CSF and the lumen [27], and macromolecules are endocytosed by lymphatic endothelial cells [16,24,27], the mechanisms by which extravasated RBCs enter lymphatic vessels are poorly understood [11,27]. Poor lymphatic drainage would prolong the exposure of brain tissue and blood vessels to free Hb and other RBC degradants, exacerbating microglial activation [27], EBI and DCI.

## 6. Multiple Possible Routes of CSF and Hb Excretion

CSF is known to drain from the subarachnoid space to the deep cervical lymphatic system and blood vessels via four major pathways (Figure 1) [26], although the pathways across the cribriform plate and along the exits of other cranial nerves, spinal nerves and blood vessels may be less active in humans than in rodents [24]. The first route is the extracellular spaces contained within the sheathes of cranial nerves, especially the olfactory nerves that lead to lymphatics (and blood microvessels) in the nasal mucosa via the cribriform plate [26]. CSF, immune cells and RBC efflux into the cribriform plate lymphatics and orbital lymphatics were observed through the subarachnoid space surrounding the olfactory and optic nerves, meaning the presence of a direct and open pathway, that is, gaps in the arachnoid membrane with no barriers for particles of 6–7 μm in size [8,11]. The other three routes are arachnoid-villi-like structures located at spinal nerve roots that connect to the meningeal ISF and/or lymphatics; arachnoid villi (containing arachnoid granulations) allowing passage across the meninges to lymphatics and venous sinuses; and perivascular or paravascular spaces leading to meningeal lymphatics along dural sinuses or at the base of the skull [26]. In addition to meningeal lymphatics within the dura mater, the human brain contains a perivascular network that allows solutes, cells and ISF to move from the brain parenchyma into the extracranial veins and lymphatic system [23].

The intramural periarterial drainage (iPAD) at the base of the skull is thought to be a pathway for the drainage of ISF from the brain to lymphatics, bypassing CSF [26]. The iPAD is a centrifugal drainage route: first, both ISF from brain tissue and CSF from the Virchow−Robin space enter the paravascular space of the basement membrane between the endothelial layer of capillaries and surrounding astrocytes, then flow into the perivascular space between the basement membranes surrounding smooth muscle cells in the tunica media of arterioles, then into the perivascular space in the adventitia of cerebral arteries, and finally into the meningeal lymphatics [16].

It has been speculated that molecules effluxing from brain tissue along the paravenous pathway (glymphatic system) may be drained into the meningeal lymphatics along cerebral veins at the brain convexities that cross the arachnoid barrier [22,23,24]. Recently, the existence of a fourth meningeal layer, the subarachnoid lymphatic-like membrane (SLYM), following the pia mater, arachnoid membrane and dura mater was reported [28], although some researchers have criticized the report [29]. SLYM separated the subarachnoid space into inner and outer compartments and fused with the pial membrane in the inner subarachnoid space, which included perivascular spaces [30]. It was reported that SLYM may form perivascular sheaths along cerebral blood vessels by encasing blood vessels and surrounding the human brain [28] and may act as a semipermeable barrier, which restricts the passage of molecules more than 1 μm in size and larger than 3 kDa in weight (that is, most peptides and proteins) and cellular blood components [28,31,32]. SLYM may also contain immune cells involved in innate defense mechanisms such as lymphatic vessel endothelial hyaluronan receptor 1 (LYVE1)-immunopositive barrier-associated macrophages [23,28,33], possibly contributing to the glymphatic system for waste excretion from the brain (Figure 1) [28,33]. Almohaimede et al. [32] examined computed tomography findings in SAH patients and reported that findings suggesting the presence of SLYM were obtained in relatively mild cases, but in severe cases with a large amount of hematoma, SLYM might have been destroyed, making it difficult to obtain findings suggesting its presence. However, current evidence for the existence of SLYM is based on histological immunophenotyping and limited imaging findings, and the macroscopic details along the neuraxis, the ultrastructure of this layer, regional structural differences (one- or two-cell-thick mesothelial membranes, impermeable or semipermeable structures), its functional role in CSF flow and the glymphatic system, and its role in the pathophysiology of diseases, including SAH, are not fully understood [32,33].

## 7. Human Glymphatic System and Meningeal Lymphatic Vessels

Many MRI techniques have been developed to image glymphatic transport in clinical settings [34]. MRI with intrathecal administration of gadolinium demonstrated the existence of the glymphatic system in the human brain [35]. It is reported that paravascular clearance activity in the human brain can be measured using an MRI technique called diffusion tensor image analysis along the paravascular space and can be calculated from the diffusivity along the deep medullary vein at the level of the lateral ventricular body [36]. Meningeal lymphatic vessels in living humans were also visualized using high-resolution gadolinium-enhanced MRI and were confirmed in autopsy, validating the presence of a central lymphatic system in humans [37]. As with animal studies, human studies showed CSF−ISF drainage through meningeal lymphatic vessels, especially along the parasagittal dura [38]. Dorsal meningeal lymphatic vessels were visualized around almost all dural sinuses, jugular veins and within the posterior aspect of the foramen magnum and were continuous with fluid channels from the skull base to the deep cervical lymph nodes [38]. Ventral dural lymphatics were found not only in the anterior cranial fossa but also at the level of cranial foramina and around the cranial nerves, with direct connections to the vascular structures and cervical lymph nodes [38]. Aging compromised the integrity of meningeal lymphatic vessels and CSF drainage, resulting in reduced clearance and atrophy of the cervical lymph nodes [38]. These discoveries overturned the traditional view that the CNS lacks lymphatic drainage and provided a new theoretical framework for studying the pathology of diseases. However, there is no direct evidence supporting that meningeal lymphatic drainage affects the glymphatic circulation in the human brain.

In patients with SAH, MRI with intrathecal gadolinium showed significant impairment of glymphatic function throughout the brain after 24 h, particularly in the cerebral cortex and subcortical white matter, which persisted for 3–6 months and remitted after 12 months [39]. When SAH patients were evaluated with MRI between two weeks and one month after the onset of SAH, those with 10 or more enlarged paravascular spaces in the basal ganglia were associated with poor 3-month outcomes, subacute hydrocephalus and DCI, while those with 10 or more enlarged paravascular spaces in the centrum semiovale were associated with poor 3-month outcomes and impaired cognitive function, suggesting a link with glymphatic system disorders [40]. In humans, the presence of paravascular blood clots was observed after aneurysmal SAH, along with enlarged paravascular spaces in the centrum semiovale and basal ganglia, suggesting an association with glymphatic dysfunction [41].

## 8. Immunohistochemical Study of Lymphoid Elements in the Human Brain

SLYM was reported to be positive for the lymphatic markers prospero homeobox protein 1 (Prox1) and podoplanin (PDPN), and the dural and arachnoid cell marker cellular retinoic acid-binding protein 2 (CRABP2), but negative for LYVE1 and the tight and adherence junction markers claudin-11 and E-cadherin [28]. These findings indicate that SLYM may be immunohistochemically distinct from the arachnoid, pia mater and lymphatics: lymphatics are positive for the classical lymphatic antigens Prox1, PDPN, LYVE1 and vascular endothelial growth factor receptor (VEGFR) 3, but negative for CRABP2 [28]. The physical rupture of SLYM may alter CSF flow patterns, causing prolonged neuroinflammation and suppressed glymphatic flows [28].

In 10 postmortem human brains with and without neurological diseases, lymphatic marker (LYVE1 and PDPN)-positive cells were present in paravascular spaces, arterial and venous walls (along the smooth muscle cell membrane and adventitia) regardless of their size, venous sinuses and between the layers of the dura mater [23]. There were also numerous lymphatic-marker-positive cells in the perineurium and endoneurium of cranial nerves, pia mater and arachnoid membrane [23]. It was hypothesized that paravascular lymphatic-marker-positive cells may originate from the pia mater and spread along the blood vessel walls from the brain surface to the deeper parenchyma [23]. However, lymphatic vessels were observed in the dura mater but not in the brain parenchyma, pia mater or arachnoid membrane [23].

## 9. Regulatory Mechanisms of Lymphangiogenesis

Understanding the regulatory mechanisms of intracranial lymphatic or CSF outflow is important, because enhancing and protecting lymphatic function improves glymphatic clearance of toxic substances and reduces neuroinflammation [14], therefore potentially mitigating EBI and DCI after SAH. The adult lymphatics are mostly quiescent, but lymphatic vessel remodeling, that is, lymphangiogenesis and/or changes in lymphatic functions, occur during some pathological conditions such as inflammation and tissue repair [42,43]. Increased lymphangiogenesis near the cribriform plate was reported during neuroinflammation and experimental autoimmune encephalomyelitis and was considered to drain excess fluid, CNS-derived antigens and cells, thus contributing to the management of neuroinflammation-induced fluid accumulation and immune surveillance [8,44]. Lymphangiogenesis may also serve as a compensatory mechanism to manage neuroinflammation-induced edema accompanied by increased numbers of aquaporin-1-positive lymphatic endothelial cells that are involved in fluid uptake [8]. However, during neuroinflammation, lymphangiogenesis was observed in the meningeal lymphatics near the cribriform plate [8], whereas during experimental autoimmune encephalomyelitis, lymphangiogenesis was observed in the lymphatics near the cribriform plate but not in the meningeal lymphatics [44].

The most potent and specific prolymphangiogenic signaling pathways involve vascular endothelial growth factors (VEGFs), particularly VEGF-C and VEGF-D through VEGFR3 [42]. During neuroinflammation, VEGF-C-producing macrophages and dendritic cells are increased and VEGF-C is upregulated near the cribriform plate to induce VEGFR3-dependent lymphangiogenesis [8,44]. Dendritic cells are largely absent from the brain parenchyma and are primarily restricted to the meninges and perivascular spaces [44]. However, migration of monocytic dendritic cells into the brain parenchyma is increased during neuroinflammation such as experimental autoimmune encephalomyelitis [44]. The migrated dendritic cells can recruit other cells, such as macrophages, to produce VEGF-C and induce lymphangiogenesis [44]. At sites of inflammation, VEGF-A/VEGFR2 signaling also promotes lymphangiogenesis through the recruitment of macrophages [43]. Other inflammation-related signaling pathways to stimulate lymphangionesis include tumor necrosis factor-α, lymphotoxin-α, Toll-like receptors, nuclear factor-κB, erythropoietin, fibroblast growth factor-2, cyclooxygenase-2 and prostaglandin E2-mediated signaling [42]. It is well known that after SAH, inflammatory responses occur via free Hb and other mechanisms, including an increase in some of the aforementioned inflammatory substances associated with lymphatic vessel proliferation [1]. However, it is unknown whether lymphatic vessel proliferation occurs after SAH. Although the information on negative regulation of lymphangiogenesis is limited, interferon-γ/Janus kinase/signal transducer and activator of transcription, transforming growth factor (TGF)-β, endostatin, semaphorin3E−PlexinD1, thrombospondin and tenascin-C (TNC)-mediated signaling have been reported as negative regulators of lymphangiogenesis [42,45].

## 10. TNC in Regulating Lymphatic Vessels

**Figure 2:**
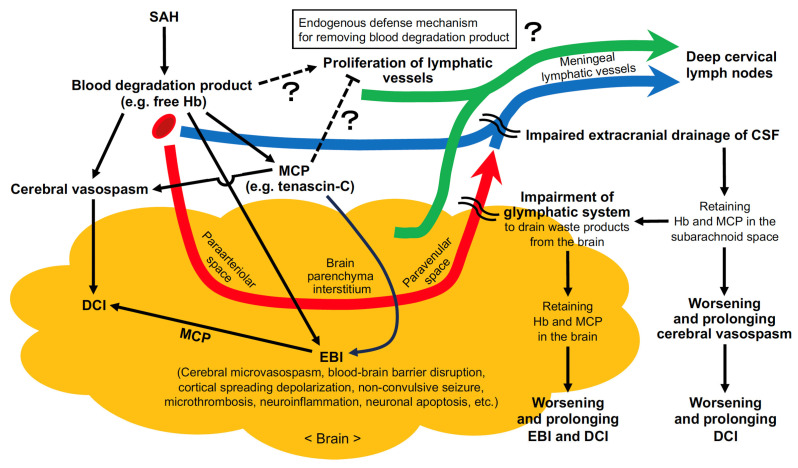
Possible mechanism of pathophysiology after subarachnoid hemorrhage (SAH). Thick red arrow, glymphatic system (impaired after SAH); thick blue arrow, lymphatic drainage from the subarachnoid space (impaired after SAH); thick green arrow, lymphatic drainage as a compensatory mechanism due to lymphatic vessel proliferation after SAH (not yet proven). CSF, cerebrospinal fluid; DCI, delayed cerebral ischemia; EBI, early brain injury; Hb, hemoglobin; MCP, matricellular protein.

A matricellular protein TNC has been reported to be a causative factor of EBI and DCI after SAH by regulating multiple signaling pathways and upregulating inflammatory molecules [46,47]. TNC was also reported to act as a spatiotemporal negative regulator of lymphangiogenesis during peripheral inflammation [45]. Genetic deletion of *Tnc* promoted lymphangiogenesis, improved lymphatic drainage function and accelerated inflammatory resolution, whereas exogenous addition of TNC suppressed lymphangiogenesis and prolonged inflammation in a mouse model of tail lymphedema [45]. The binding of TNC to the integrin αvβ1 in lymphatic endothelial cells directly inhibited lymphangiogenesis induced by prolymphangiogenic factors such as fibroblast growth factor-2 and VEGF-C by activating p38 mitogen-activated protein kinase via Smad-independent TGF-β signaling pathways in mouse skin [45]. However, the relationship between TNC and lymphangiogenesis has only been demonstrated in extracranial organs, and there is no direct evidence of TNC-mediated regulation of intracranial lymphatic vessels in SAH models. As TNC is upregulated intracranially during the acute phase of SAH [46], the authors hypothesize that the mechanism of TNC-induced EBI and DCI may involve its inhibitory effect on lymphatic vessels. Because TNC has also been suggested to be associated with other matricellular proteins that are upregulated after SAH and involved in the pathogenesis of EBI and DCI [48], the relationship between other matricellular proteins and lymphatic drainage after SAH is also of interest (Figure 2).

## 11. Possible Roles of Platelets in Regulating Lymphatic Vessels

After SAH, many molecules and mediators such as Hb degradation products, platelets, complements, cytokines, chemokines, matricellular proteins and inflammatory cells are biologically active within the CSF and have been considered to be involved in secondary pathophysiological events [49,50]. For example, platelets extravasated after SAH may play a role in the development of microthrombi near cerebral arteries, inflammatory reactions and vasospasm [49]. Recently, it has been reported that the hemostatic system (platelets, coagulation and fibrinolysis) plays various pathophysiological roles in lymphangiogenesis [51]. Platelets are required for separating lymph sacs from the cardinal veins via C-type lectin-like receptor-2/PDPN signaling in lymphatic endothelial cells under some pathological conditions [51]. Platelet-derived VEGF-C activation by thrombin and plasmin may promote lymphangiogenesis, while TGF-β1, which is abundant in platelets, may have antilymphangiogenic effects [52,53]. Platelet extracellular vesicles were also reported to maintain the integrity of lymphatic endothelial cells and enhance lymphatic function in mice [54]. Like coagulation and fibrinolysis, platelet activation is a physiological phenomenon that inevitably occurs in the CSF space after SAH. Therefore, platelets are expected to affect intracranial lymphatic vessels after SAH, but this has not been investigated to date.

## 12. Possible Changes in Lymphatics After SAH and Other Strokes

There have been several reports in animal models regarding lymphangiogenesis after stroke [55,56,57,58]. After intracerebral hemorrhage, lymphangiogenesis has been reported to contribute to hematoma resolution and to have a neuroprotective effect [57,58]. After cerebral infarction, however, there are conflicting reports, with some reporting that lymphangiogenesis had a neuroprotective effect [56] and others reporting that it exacerbated brain damage [55]. It is hypothesized that waste products leaving the brain and entering the CSF are excreted through lymphatic capillaries in the subarachnoid space that branch off from the meningeal lymphatics [59], which may be increased by day 3 to promote blood clearance by simvastatin treatment in a rat model of intracerebral hemorrhage [57]. Increased meningeal lymphangiogenesis and lymphatic drainage occurred from days 10 to 14 after intracerebral hemorrhage in mice, and administration of cilostazol accelerated lymphangiogenesis by day 3 and promoted RBC clearance [58]. In a mouse model of transient middle cerebral artery occlusion, VEGF-C/VEGFR3 signaling induced lymphangiogenesis exclusively near the cribriform plate, beginning by day 3, peaking at day 7, and regressing by day 14 [55]. In this model, acute lymphangiogenesis was shown to exacerbate cerebral infarction [55]. In a recent study, VEGF-C administration reduced neurological deficits and inflammation in a mouse model of transient middle cerebral artery occlusion [56]. The findings were accompanied by increased proliferation of lymphatic capillaries that branched off from meningeal lymphatics around areas such as the olfactory bulb, cerebral cortex and cerebellum, as well as increased drainage of brain ISF and CSF (Figure 1) [56]. To our knowledge, no clinical studies have been conducted on intracranial lymphatic changes in cerebrovascular disorders.

After SAH, persistent impairment of lymphatic drainage and the glymphatic system are common, possibly due to obstruction by blood clots [1]. Aging also causes impairment of the glymphatic system [25]. However, it is unclear whether intracranial lymphangiogenesis or lymphangiectasia occurs after SAH as a compensatory mechanism for post-SAH CSF circulatory disorders, especially in the elderly. Post-SAH increased lymph flow in the meningeal lymphatics may not be associated with significant lymphangiogenesis and lymphangiectasia [27]. However, Hb-scavenging strategies to reduce its toxicity should include enhancing glymphatic and lymphatic function to improve the clearance of RBCs, their degradation products and RBC-laden phagocytes. Experimental studies reported that an intrathecal tissue plasminogen activator and the selective α-2-adrenergic agonist dexmedetomidine were effective for enhancing glymphatic function by facilitating blood clearance in the intraparenchymal paravascular spaces and by inducing a slow-wave sleep-like state, respectively [13,25]. Glymphatic function may also be improved by increasing arterial pulsatility, a key force driving glymphatic flow, with vasopressors such as dobutamine and by avoiding frequent neurological checks that interrupt deep non-rapid eye movement sleep, when glymphatic function is most active [25]. Manipulation of lymphatic drainage can influence disease outcomes including not only chronic disorders such as cognitive dysfunction during aging and Alzheimer’s disease, but also acute diseases such as stroke, highlighting the significance of meningeal lymphatics in the drainage of fluid, antigens and cells [8].

## 13. Current Situation of Chronic Disorders

It has been pointed out that when the glymphatic system does not function properly, it is associated with neurodegenerative diseases such as Alzheimer’s disease [6]. Research into the glymphatic system is currently progressing, but it will take some time before the results can be widely applied in general medical settings [60]. Clinically, some surgeons perform deep cervical lymphaticovenous anastomosis, an innovative microsurgical technique aimed at promoting drainage of brain waste products by anastomosing deep cervical lymphatic vessels to adjacent veins [61]. This procedure is expected to promote the removal of amyloid beta and tau through the drainage pathways of the brain, meninges and deep cervical lymphatic vessels, improving cognitive function in patients with Alzheimer’s disease [61]. However, there is still no evidence of its effectiveness, and this procedure may only have potential benefits if the lymphatic pathways in the brain parenchyma and meninges are not substantially blocked [61]. As a result, it is currently difficult for hospitals that treat chronic diseases to provide specialized treatment in this field.

## 14. Future Directions

As mentioned above, new findings regarding CSF circulation have been emerging in recent years, but little is known about the relationship between intracranial lymphatics and brain injury, particularly EBI and DCI after SAH. This may be because researchers have focused on inhibiting the series of reactions that cause brain injury, and methods for controlling CSF circulation and lymphatic drainage have been limited. SAH is likely to involve SLYM rupture upon cerebral aneurysm rupture, and free Hb diffuses widely into the CSF cavity. Therefore, compared with other pathological conditions, SAH is expected to have a greater impact on CSF circulation, intracranial lymphatics and brain injury; however, this is not fully understood. Elucidating these findings will not only lead to the development of new treatments for EBI and DCI after SAH, such as controlling intracranial lymphatic vessel proliferation, but will also constitute important research that will point in new directions for the development of prevention and treatment strategies for hydrocephalus and higher brain dysfunction after stroke.

## Data Availability

No new data were created or analyzed in this study.

## Abbreviations

BBB, blood−brain barrier; CNS, central nervous system; CRABP2, cellular retinoic acid-binding protein 2; CSF, cerebrospinal fluid; DCI, delayed cerebral ischemia; EBI, early brain injury; Hb, hemoglobin; Hp, haptoglobin; ICP, intracranial pressure; IgG, immunoglobulin G; iPAD, intramural periarterial drainage; ISF, interstitial fluid; MRI, magnetic resonance imaging; PDPN, podoplanin; Prox1, prospero homeobox protein 1; RBC, red blood cell; SAH, subarachnoid hemorrhage; SLYM, subarachnoid lymphatic-like membrane; TGF, transforming growth factor; TNC, tenascin-C; VEGF, vascular endothelial growth factor; VEGFR, vascular endothelial growth factor receptor.
